# Supplementary material for: Molecular Studies of the Protein Complexes Involving Cis-Prenyltransferase in Guayule (Parthenium argentatum), an Alternative Rubber-Producing Plant
Source: Front Plant Sci. 2019 Feb 25;10:165. doi: 10.3389/fpls.2019.00165 (PMC6397875; doi:10.3389/fpls.2019.00165)
Supplement: Supplementary file 1 [file Data_Sheet_1.docx]

| **Supplemental Table 1. List of primers used in this work.** | | |
| --- | --- | --- |
| No. | Primer Name | Sequences |
| 1 | PaCPT1-F | GAATCATAGGTAAGTGTGGAAATTGGTATG |
| 2 | PaCPT1-R | AGTTGAATCGATTCATGTCTGCTTCTGTTTGTTTAA |
| 3 | PaCPT1-P414-F | AGTGTCACTAGTCCAACATGGATATTAAACAGAGAACTCGAATATACAACA |
| 4 | PaCPT1-P414-R | GACAGTGTCGACTCATGTCTGCTTCTGTTTGTTTAAATAATACA |
| 5 | PaCPT2-P414-F | CCGTAACTAGTAACATGGAAGAAGAAAGACC |
| 6 | PaCPT2-P414-R | ACGATCCCGGGCTCGAGTTA |
| 7 | PaCPT3-P414-F | AGTGTCGGATCCACACCATGGAAGTCAACCCAATTATCACCAC |
| 8 | PaCPT3-P414-R | GACAGTATCGATTTAAGCTTGCTTCTTCTTCTTTTCCAAG |
| 9 | PaCBP-Bait-C-F | AGTGTAAGGCCTCCACGATGGATTTGGTCGCCGAATCTC |
| 10 | PaCBP-Bait-C-R | GACAGTCCATGGAATCAGGAACCGTAGTTTTGTTTGACCT |
| 11 | PaCPT1-prey-C-F | AGTGTCACTAGTCCAACATGGATATTAAACAGAGAACTCGAATATACAACA |
| 12 | PaCPT1-prey-C-R | GACAGTGGATCCGTGTCTGCTTCTGTTTGTTTAAATAATACAATTTATG |
| 13 | PaCPT2-prey-C-F | AGTGTCGGATCCACACCATGGAAGAAGAAAGACCATCCGGT |
| 14 | PaCPT2-prey-C-R | GACAGTATCGATAGCTTGCTTCTTCTTCTTTTCCAAGTAGTA |
| 15 | PaCPT3-Prey-C-F | AGTGTCGGATCCACACCATGGAAGTCAACCCAATTATCACCAC |
| 16 | PaCPT3-Prey-C-R | GACAGTATCGATAGCTTGCTTCTTCTTCTTTTCCAAGTAC |
| 17 | PaCPT1-prey-N-F | AGTGTCGGATCCATGGATATTAAACAGAGAACTCGAATATACAACA |
| 18 | PaCPT1-prey-N-R | GACAGTGTCGACTCATGTCTGCTTCTGTTTGTTTAAATAATACA |
| 19 | PaCPT2-prey-N-F | AGTGTCGGATCCATGGAAGAAGAAAGACCATCCGGT |
| 20 | PaCPT2-prey-N-R | GACAGTATCGATTTAAGCTTGCTTCTTCTTCTTTTCCAA |
| 21 | PaCPT3-Prey-N-F | AGTGTCGGATCCATGGAAGTCAACCCAATTATCACCAC |
| 22 | PaCPT3-Prey-N-R | GACAGTATCGATTTAAGCTTGCTTCTTCTTCTTTTCCAAG |
| 23 | IRES forward | AGAAGGTACCCCATTGTATGGGATC |
| 24 | FLAG 1 reverse | CTTTATAATCACCGTCATGGTCTTTGTAGTCGGCCATATTATCATCGTGTTTTTC |
| 25 | FLAG 2 reverse | GTCATCGTCATCCTTGTAATCGATATCATGATCTTTATAATCACCGTCATGGTCTTTG |
| 26 | FLAG 3 reverse | ATTCGGATCCCATATGGGTGGTGGCCAGCTTGTCATCGTCATCCTTGTAATCGATATC |
| 27 | PaCPT3 forward | TGGCCACCACCCATATGGGATCCATGGAAGTCAATCCAATCATCACA |
| 28 | PaCPT3 reverse | TTTTTTTTTTTTCAGTCAGATCTCACTCGAGTCAAGCCTGCTTCTTCTTCTTCTC |
| 29 | PaCBP forward | ATTAGGATCCATGGATTTGGTCGCCGAATC |
| 30 | PaCBP reverse | ATTACTCGAGTCAGGAACCGTAGTTTTGTTTGAC |
| 31 | GFP forward | ATATGGATCCATGGAGAGCGACGAGAGCG |
| 32 | GFP reverse | ATATCTCGAGTCATTCTTCACCGGCATCTGC |


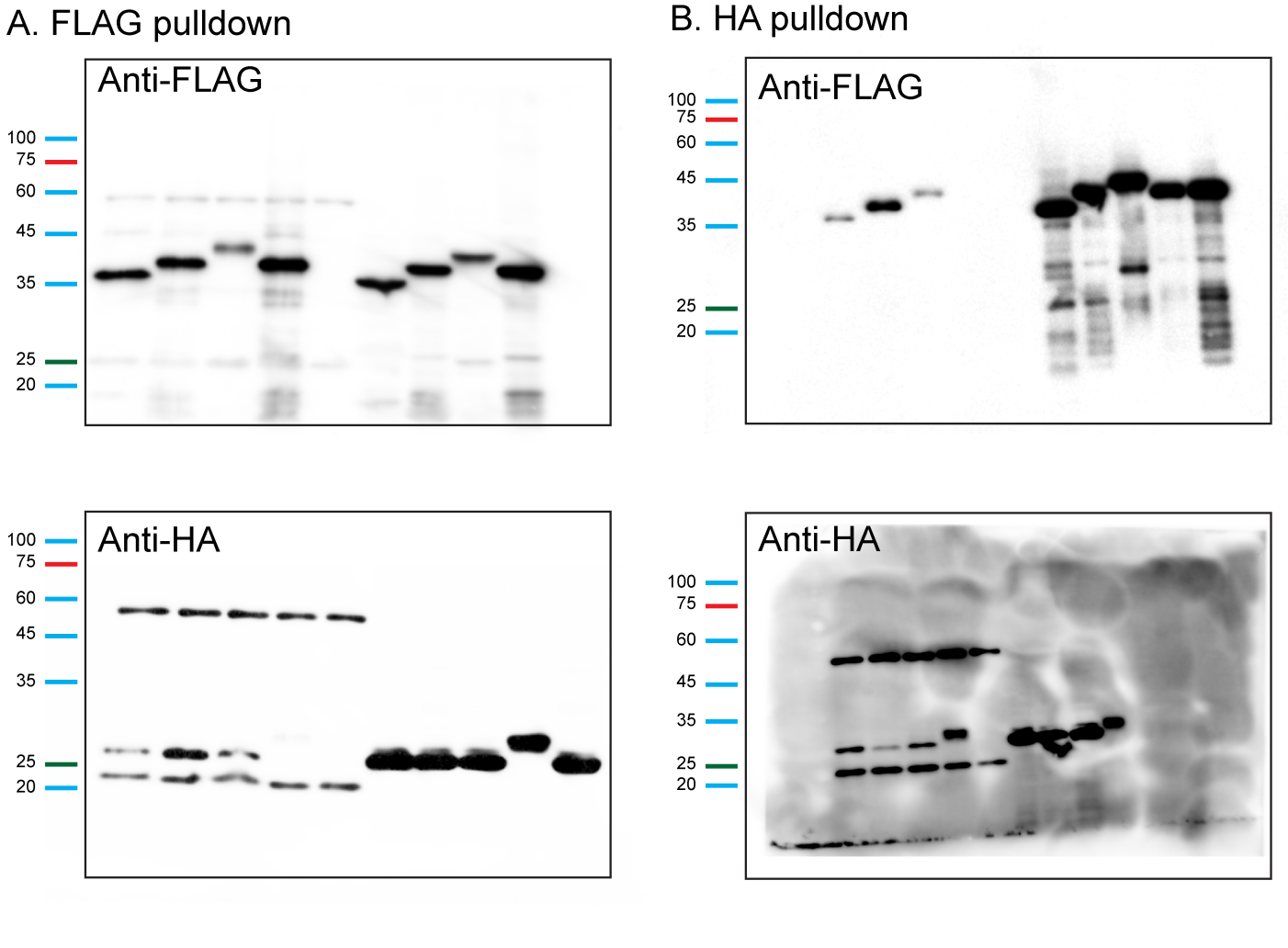


**Supplemental Figure 1. Raw image data of co-immunoprecipitation experiments (Figure 5 in the main text)**
